# Impact of commercial gut health interventions on caecal metagenome and broiler performance

**DOI:** 10.1186/s40168-024-02012-7

**Published:** 2025-01-29

**Authors:** Gladys Maria Pangga, Banaz Star-Shirko, Androniki Psifidi, Dong Xia, Nicolae Corcionivoschi, Carmel Kelly, Callie Hughes, Ursula Lavery, Anne Richmond, Umer Zeeshan Ijaz, Ozan Gundogdu

**Affiliations:** 1 Faculty of Infectious and Tropical Diseases, London School of Hygiene and Tropical Medicine, London, UK; 2 Royal Veterinary College, London, UK; 3 Bacteriology Branch, Agri-Food and Biosciences Institute, Veterinary Sciences Division, Belfast, UK; 4 Faculty of Bioengineering of Animal Resources, University of Life Sciences King Mihai Timișoara, Timișoara, Romania; 5 Pilgrim’s Europe Ltd, Craigavon, UK; 6 James Watt School of Engineering, University of Glasgow, Glasgow, UK

**Keywords:** Chicken gut microbiome, Metagenomics, Gut health, Broiler performance, Shotgun sequencing, Probiotics

## Abstract

**Background:**

Maintaining gut health is a persistent and unresolved challenge in the poultry industry. Given the critical role of gut health in chicken performance and welfare, there is a pressing need to identify effective gut health intervention (GHI) strategies to ensure optimal outcomes in poultry farming. In this study, across three broiler production cycles, we compared the metagenomes and performance of broilers provided with ionophores (as the control group) against birds subjected to five different GHI combinations involving vaccination, probiotics, prebiotics, essential oils, and reduction of ionophore use.

**Results:**

Using a binning strategy, 84 (≥ 75% completeness, ≤ 5% contamination) metagenome-assembled genomes (MAGs) from 118 caecal samples were recovered and annotated for their metabolic potential. The majority of these (*n* = 52, 61%) had a differential response across all cohorts and are associated with the performance parameter — European poultry efficiency factor (EPEF). The control group exhibited the highest EPEF, followed closely by the cohort where probiotics are used in conjunction with vaccination. The use of probiotics B, a commercial *Bacillus* strain-based formulation, was determined to contribute to the superior performance of birds. GHI supplementation generally affected the abundance of microbial enzymes relating to carbohydrate and protein digestion and metabolic pathways relating to energy, nucleotide synthesis, short-chain fatty acid synthesis, and drug-transport systems. These shifts are hypothesised to differentiate performance among groups and cycles, highlighting the beneficial role of several bacteria, including *Rikenella microfusus* and UBA7160 species.

**Conclusions:**

All GHIs are shown to be effective methods for gut microbial modulation, with varying influences on MAG diversity, composition, and microbial functions. These metagenomic insights greatly enhance our understanding of microbiota-related metabolic pathways, enabling us to devise strategies against enteric pathogens related to poultry products and presenting new opportunities to improve overall poultry performance and health.

Video Abstract

**Supplementary Information:**

The online version contains supplementary material available at 10.1186/s40168-024-02012-7.

## Background

The gastrointestinal tract of chickens harbours a complex and dynamic microbial community collectively known as the gut microbiota. This microbiota, along with its corresponding genetic material, forms the gut microbiome, which is recognised for its significance in both health and metabolism in its host [1]. The majority of the microbiome consists of a diverse set of bacteria which can be classified as either commensal, pathogenic, or beneficial to the host, of which a variety of factors, such as genetics, age, environment, diet, and administration of feed additives, can influence their occurrence and interactions [1–4]. Interestingly, these same factors have also been established to directly impact the overall health and performance of poultry [5–7], indicating a possible link between gut microbiota composition and bird performance. For example, a study identified 24 bacterial species to be differentially abundant between broilers with high and low feed conversion ratios (FCR) [8]. Furthermore, in our previous work, we demonstrated that extrinsic parameters, including stocking density, percentage of protein and energy in the diet, and omega-3 supplementation, can modulate key microbiome members involved in nutrition and metabolism, subsequently affecting growth and feed efficiency in the host [9].

Due to this accumulating evidence supporting the importance of gut health in poultry performance, there has been a significant rise in interest in the modulation of gut microbiota for improved animal health, productivity, and food safety [10]. Historically, growth promoters have been utilised for enhanced feed efficiency while also decreasing illness and death rates from both overt and hidden diseases [11]. These drugs are purported to achieve these benefits by altering the gut microbiota, resulting in decreased nutrient utilisation by microbes, increased absorption of nutrients through thinned host gut walls, and reduction in inflammatory stress [11–13]. Ionophores are growth promoters that have been safely used as the approved and standard intervention to maintain gut health in poultry in the last decades, especially for the effective prevention of economically important diseases such as coccidiosis and necrotic enteritis [14–16]. Currently, a myriad of new gut health interventions (GHIs) with similar effects have emerged for further improvement of bird health and performance, which can be generally represented by the European poultry efficiency factor (EPEF), a metric that integrates performance information on weight gain, feed conversion, and mortality [17, 18]. GHIs include but are not limited to prebiotics, probiotics, phytogenic substances, organic acids, essential oils, and enzymes [19]. Each of these GHIs has its mechanism of action and corresponding effects on chickens. Descriptions of these GHIs have been detailed previously in recent reviews [20, 21]. Options are further expanded by the combinatory use of multiple GHIs throughout a single production cycle for their potential synergistic effects [22, 23]. However, there is limited information on their effects on the gut microbiome in poultry.

Research investigating the influence of GHIs has primarily involved the use of metataxonomic sequencing of the microbiota through amplification of the 16S rRNA gene marker [24]. This approach has proved ground-breaking for our understanding of gut health. For instance, Robinson et al. [25] documented increases in alpha-diversity evenness and suppression of certain microbial members of the Firmicutes phylum in the gut of birds administered with ionophores such as salinomycin and monensin. Probiotics, on the other hand, were observed to enhance the level of Tenericutes members and activate various energy-related pathways in the broiler caeca [26]. However, taxa identification of less abundant and unknown species, as well as the characterising of metabolic capacity (functional profiles) of individual gut microorganisms, remains challenging [24, 27, 28]. By contrast, with shotgun sequencing (which involves indiscriminate sequencing of all random DNA segments within a sample), a higher resolution of microbial genomes enables these features to be identified, allowing a deeper understanding of the relevant metabolic functions of each gut microorganism [28]. For example, Chen et al. (2023) [29] used shotgun metagenomics to elucidate the role of gut microbiota in fat regulation in chickens, uncovering differences in carbohydrate-active enzymes (CAZymes) and functional metabolic modules between chickens with high and low abdominal fat. Building on this concept, an exploration of the metagenome of chickens given various GHIs can reveal functional insights into the microbial breakdown of carbohydrates, protein, and other macromolecules in the gut. This, in turn, could enhance our understanding of nutrient absorption processes in broilers, supporting the optimisation of current feed management strategies. Therefore, this study aims to characterise how GHIs impact bird performance, the gut microbiome, and its role in feed metabolism through shotgun metagenomic sequencing in comparison to the standard use of ionophores.

## Methods

### Ethics statement

Poultry farm management and industry plant processing activities were conducted following Pilgrim’s Europe Ltd. (formerly Moy Park Ltd.) standard operating protocols, which are compliant with UK animal handling laws and regulations [30, 31]. As part of the standard commercial practices of the company, all birds were subjected to electrical stunning before slaughter and subsequent carcass processing [32].

### Experimental design and sampling

**Fig. 1:**
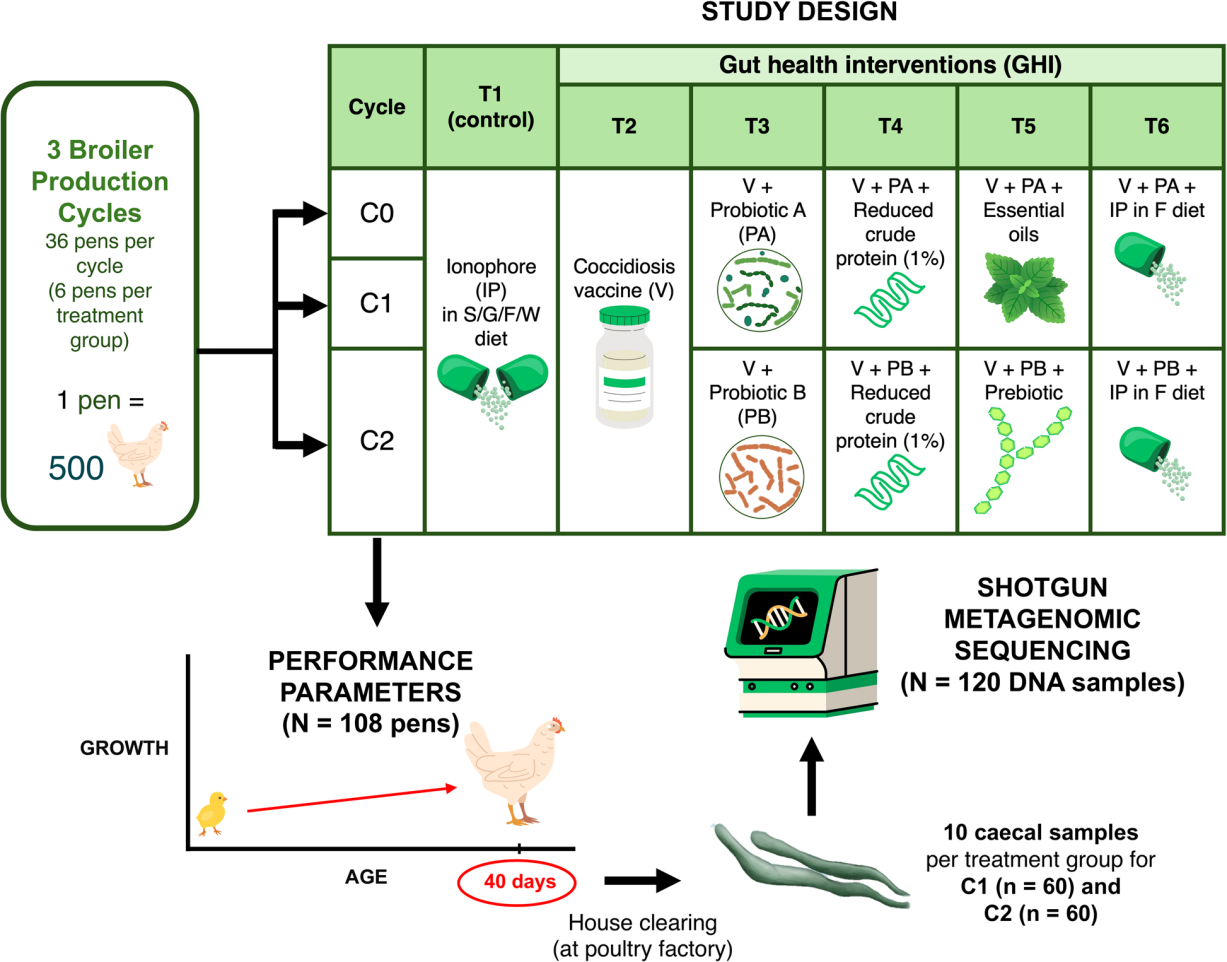
Overview of study design. C, production cycle; S, starter; G, grower; F, finisher; W, withdrawal; PA, probiotic A; PB, probiotic B; IP, ionophore; V, coccidiosis vaccine

Three broiler production cycles (C0, C1, C2) were implemented in an automated commercial poultry house, which was described previously by Hanna et al. [33] between June and November 2022. In each cycle, a total of 18,000 Ross-308 mix-sexed broilers were raised for 40 days and were provided with a four-stage standard commercial diet regimen based on Aviagen specifications for Ross broilers [34]. This was composed of a starter diet (S, 0–11 days), a grower diet (G, 11–23 days), a finisher diet (F, 23–31 days), and a withdrawal diet (W, 32 days until clearing at day 40). Birds were distributed into 6 groups (1 control, 5 GHI groups), wherein each group was allocated to 6 pens containing 500 birds each, maintaining a stocking density not exceeding 38 kg/m^2^. For our control group, we administered ionophores, which is a safe and legally accepted method for the control of coccidiosis [15, 16]. For our treatment groups, we adapted different gut health strategies, which involved a combination of GHIs; this was designed to optimise and maximise the differences in gut health and performance as based on the study of Granstad et al. [22]. GHI treatment groups for C0 and C1 included the following: T2 — coccidiosis vaccine (V); T3 — V + *Bacillus* strain probiotics A (PA); T4 — V + PA + reduced crude protein (− 1%) in G/F/W diets; T5 — V + PA + essential oil; and T6 — V + PA + ionophore in F diet. Birds in C2 had a similar treatment design with two differences: a different GHI — *Bacillus* strain probiotics B (PB) was utilised instead of PA in T2 to T5, and essential oils in T5 were replaced by prebiotics (Illustrated in Fig. 1). Birds were provided ad libitum access to feed and water. We followed the experimental set-up of pens and bird management protocols as described previously [33]. A 10-day downtime was maintained between cycles, during which a rigorous cleaning of the house was performed, involving complete litter removal and fumigation.

At the end of the production cycle (day 40), intact paired caeca of 10 randomly selected broilers from each treatment group of C1 (*n* = 60) and C2 (*n* = 60) were collected within 5–10 min of the slaughtering process in a Pilgrim’s Europe Ltd. industry plant (Fig. 1). Caecal samples were obtained through an aseptic incision from the rest of the GIT and were transferred into sterile 50-ml tubes and stored in a polystyrene container with ice packs. All specimens were immediately sent to the laboratory for storage at − 80 °C until used for DNA extraction.

### DNA extraction, library preparation, and shotgun metagenomic sequencing

Microbial DNA was extracted from caecal content using the QIAamp® PowerFecal Pro DNA Kit (Qiagen, Germany) according to the manufacturer’s instructions. For each sample, 200–250 µg of caecal content was aseptically collected from a randomly selected caecal pouch. The caecal content and 800 µl of lysis buffer were then added to a bead-beating tube and vortexed at maximum speed for 10 min. After centrifugation, the supernatant was transferred to a clean tube, mixed with solutions for inhibitor removal and DNA binding, and then loaded onto spin columns. Each column was washed twice to remove non-DNA contaminants, eluted in 100 µl of elution buffer, and centrifuged to collect purified DNA, which was then stored at − 20 °C. Initial DNA concentration was also measured using NanoDrop ND-1000 (NanoDrop Technologies, Inc., Wilmington, USA).

Sequencing libraries were generated using a modified Illumina DNA Prep tagmentation approach (Illumina, Inc., Cambridge, UK) described previously [35]. Tagmentation was performed as follows: a master mix composed of 0.5-µl bead linked transposomes, 0.5-µl tagmentation buffer, and 4-µl nuclease-free distilled water was created for each sample (2 µl), placed in a 96-well plate, and run on a thermocycler at 55 °C for 15 min. Another PCR master mix using the KAPA2G Fast HotStart PCR Kit (Sigma-Aldrich, Gillingham, UK) was then generated and transferred into a 96-well plate, to which 5 µl of P7 and P7 of Nextera XT Index Kit v2 index primers (Illumina, Cambridge, UK) and 7 µl of the previous tagmentation reaction were added. The plate was run on the thermocycler with conditions: 72 °C for 3 min, 95 °C for 1 min, 14 cycles of 95 °C for 10 s, 55 °C for 20 s, and 72 °C for 3 min. Quality control of multiplex barcoding was performed on a D5000 ScreenTape using the Agilent Tapestation 4200 (Agilent, Waldbronn, Germany). Next, barcoded libraries were quantified on a Qubit 3.0 instrument (Invitrogen, Paisley, UK), pooled in equivalent concentrations in a tube, and washed with 0.5–0.7X solid-phase reversible immobilisation KAPA Pure Beads (Roche, Wilmington, USA). To calculate the final pool molarity, the pooled library was quantified using Qubit 3.0 and on a D5000 High Sensitivity ScreenTape. After library qualification, the library was sequenced using the NovaSeq 6000 System, paired-end 150 bp (Illumina, Cambridge, UK).

### Bird performance and health monitoring

The performance parameters included bird weight (BW, kg of body weight), average daily gain (ADG, grams feed per day), corrected feed conversion ratio (FCR) at 2-kg BW, and total mortality in % (MT). These measurements were taken as mean average per pen at clearing day (day 40), which were conducted in line with typical industrial practices. Contact dermatitis measures, which included footpad dermatitis (FPD), lesion scores (FPDS), FPD prevalence (%, FPDP), and hock burn (HB) lesion scores (HBS) and prevalence (%, HBP), were also taken before slaughter as conducted previously [5, 33]. To estimate overall health and performance, the European production efficiency factor (EPEF) was calculated as previously described [36].

## Bioinformatic analysis

### Recovery of metagenomic-assembled genomes (MAGs)

A total of 120 metagenomic samples were processed — from which adapter-trimmed reads from two lanes were generated by the sequencing centre. Reads were merged and subjected to quality trimming using Sickle v1.200 [37]. Trimming involved removing reads where the average phred was below 20 and retaining paired-end reads with a post-trimming length exceeding 50 bp. Two samples (1 from T3 and 1 from T4 in C2) were excluded due to non-recovery of reads, resulting in a total of 118 samples which generated 2,588,938,595 reads. Forward and reverse reads were then aggregated and subjected to collective assembly using MEGAHIT [38]. Assembly parameters used were –k-list 27,47,67,87 –kmin-1pass -m 0.95 –min-contig-len 1000. This gave us a total of 1,276,325 contigs, a total of 3,101,580,806 bases (bp), a maximum of 403,439 bp, an average length of 2430 bp, and an N50 score of 2724 bp. Assemblies were then subjected to binning via the MetaWRAP pipeline [39], wherein three algorithms, namely MetaBAT 2 [40], MaxBin [41], and CONCOCT [42], were utilised. Bins from each of the algorithms were consolidated using the MetaWRAP framework, resulting in a total of 308 bins. For the estimation of completion (COM) and contamination (CON) metrics of each MAG, CheckM was used on all bins [43]. We retained bins with more than 75% and less than 5% contamination to give a final set of 84 MAGs. The summary statistics of these MAGs are provided in Supplement File 1.

### Taxonomic and functional annotation

For metabolic function and taxonomic assessment of each MAG, the METABOLIC pipeline was employed [44]. Within its framework, the taxonomic classification of bins was incorporated using GTDB-Tk [45], while functional annotations were recovered using Kyoto Encyclopedia of Genes and Genomes (KEGG) for metabolic function modules and submodules [46], dbCAN2 for carbohydrate-active enzymes (CAZymes) [47], custom hidden Markov model databases for nutrient cycles [48], and MEROPS for proteases [49]. To obtain taxonomic and functional coverages per sample, read coverages (proportion of each bin per sample) were multiplied with each feature coverage (returned from METABOLIC). From this, we derived the sample-wise abundance tables: dbCAn2 (*n* = 118 samples × 117 CAZyme IDs), KEGG modules (*n* = 118 samples × 251 module IDs), KEGG submodules (*n* = 118 samples × 964 submodule IDs), and MEROPS (*n* = 118 samples × 108 peptidases).

### Phylogenetic tree generation

To construct a phylogenetic tree of MAGs, we used GToTree [50], which involves the detection of single-copy genes (SCGs) in MAGs and multisequence alignment. Specifically, we used the bacteria and archaea HMM set, which covers 25 SCGs. MAGs that had very few hits for these SCG were removed, resulting in a phylogeny recovery for a total of 65 MAGs. For assessment of the novelty of MAGs, the Genome Tree Toolkit was utilised [51], wherein phylogenetic gain (PG) for each MAG against other MAGs in the tree was estimated.

### Statistical analyses

All tests were performed in R [52]. The normality of data was assessed using the Shapiro–Wilk test [53]. To determine significant differences between treatment groups, we employed analysis of variance (ANOVA) and pairwise *t*-test with Bonferroni correction for normally distributed data while Kruskal–Wallis Rank Sum testing and post hoc Dunn testing with Bonferroni correction (*p* < 0.05) for non-normal-distributed data [52]. For performance data, when significant *p*-values (*p* < 0.05) were obtained in ANOVA, statistical groupings were evaluated using the Duncan’s multiple range test (DMRT) through the *agricolae* package [54].

To evaluate the individual effects of GHIs across the three cycles, we performed a generalised linear model analysis via the penalised maximum likelihood method using the *glmnet* package [55]. We applied the least absolute shrinkage and selection operator (LASSO) model, with alpha set to 1 and tenfold cross-validation. This approach helped prevent overfitting caused by multicollinearity and sparse covariates (such as vaccination and probiotic use in our study) while identifying the best predictors of the outcome of interest. The model is represented as follows: $minimise\left({\sum }_{i=1}^{N}{\left({y}_{i}-{\beta }_{0}-{\sum }_{j=1}^{p}{x}_{ij}{\beta }_{j}\right)}^{2}+\lambda {\sum }_{j=1}^{p}\left|{\beta }_{j}\right|\right)$, where $N$: the number of observations, $p$: the number of predictors, ${y}_{i}$: the outcome variable for the $i$-th observation, ${x}_{ij}$: the value of the $j$-th predictor for the $i$-th observation, ${\beta }_{0}$: intercept term, ${\beta }_{j}$: the coefficients for each predictor, and $\lambda$: the regularisation parameter controlling the strength of the penalty term [56]. This model indicates that the penalty term forces some of the beta coefficients to go to zero when their corresponding predictors are not significant. For our model, we included the following as predictors: ionophore (all stages) as “yes” or “no”; ionophore (finisher only) as “yes” or “no”; vaccination: “yes” or “no”; probiotic A: “yes” or “no”; probiotic B: “yes” or “no”; essential oil: “yes” or “no”; reduced crude protein (− 1%): “yes” or “no”; and prebiotic: “yes” or “no”, wherein “no” was used as a reference for all covariates. For outcome variables, we used the parameters 40-day ADG, 40-day BW, EPEF, corrected 2-kg FCR (2-kg FCR), FPDP (%), HBP (%), and total MT (%).

For microbial diversity assessment, different functions of the *vegan* package [57] were employed. For alpha diversity, we estimated richness (R) (using the *rarefy* function), Shannon entropy (H) and Simpson (Si) (using the *diversity* function), Fisher alpha (FA) (using the *fisher.alpha* function), and Pielou’s evenness (PE) (using the *specnumber* (S) function for formula: PE $=\frac{H}{\text{log}(S)}$). After confirming their normal distribution, ANOVA was then employed to determine significant differences between treatment groups [52]. For beta diversity, we employed Bray–Curtis dissimilarity index analysis using the *vegdist* function of the *vegan* package, followed by principal component analysis using the R base function *cmdscale* [52, 57]. The separation between treatment groups and between cycles was tested with permutational analysis of variance (PERMANOVA) through the *vegan* command *adonis* [57]*.*

To find the relationship between the individual MAGs and each treatment group (C1 and C2 performed separately) as well as the relationship between individual MAGs and performance parameters — EPEF and MT, we employed a generalised linear latent variable model (GLLVM) regression analysis as described previously [58]. In this model, the mean abundances (for the *i-*th sample and *j-*th MAG) were regressed against sources of variation (${x}_{i}$, treatment groups or performance/health traits) by incorporating latent variables ${u}_{i}$ (which are confounders and not observed) as follows: $g\left({\mu }_{ij}\right)={\eta }_{ij}={\alpha }_{i}+{\beta }_{0j}+{x}_{i}^{T}{\beta }_{j}+{u}_{i}^{T}{\theta }_{j}$, where ${\beta }_{j}$ are covariate-specific coefficients which, if significant, aid in understanding the role of the MAG with respect to that covariate. Note that the 95% confidence intervals of these coefficients were generated, and where they crossed the 0 boundary, they were deemed insignificant. We conducted this GLLVM analysis through the *gllvm* package, where we specified the use of the negative binomial distribution and the variational approximation method [59].

For comparison of grouped features between treatment groups, sample-wise abundance tables were initially subjected to normalisation by total sum scaling (TSS) (number of reads for each MAG divided by total of reads per sample) and subsequently via centralised log ratio (CLR) method using the *logratio.transfo* function (log-ratio transformation) of the *mixOmics* package [60]. Meanwhile, for individual feature statistical comparison between treatment groups, sample-wise abundance tables were subjected to differential expression analysis using the *DESeq2* package with default settings (test: negative binomial Wald test, type of fitting of dispersions to the mean intensity: *parametric*, with *p* < 0.05) [61]. Individual CAZyme function categorisation was adapted from dbCAN2 annotated substrate information [47] and ontology of MetaCyc database [62], with supplementing information from the CAZy database [63] and CAZypedia [64]. KEGG orthology (KO) codes used for enzyme mapping of production pathways of short-chain fatty acids (SCFA): acetate, butyrate, and propionate were adapted from previous metagenomic studies [65, 66]. Identified KO codes were then matched to KEGG submodules using the KEGG database [46].

For visualisation, *ggplot2* was used for the generation of plots (line graphs, bar plots, boxplots, Sankey plots) [67], while *ComplexHeatMap* was utilised for heatmap clustering (we used Euclidean clustering) [68]. For mapping a phylogenetic tree, we utilised the packages *ape* and *ggtree* for manipulation and layering of other MAG features: guanosine-cytosine (GC) content, novelty (PG), and quality score (computed using formula: COM — 5 × CON) [69, 70].

## Results

### Administration of different GHIs impacted overall bird performance

Growth performance is one of the most important indicators of nutrition and health in poultry. As shown in Table 1, significant differences between treatment groups were only observed in FCR, EPEF, and HB measures (ANOVA, *p* < 0.05). Estimation of corrected FCR of C0 and C2 had shown T1 to have the best FCR. EPEF was observed to be highest in T1, followed by T3, but was not significant in C0 and C1. Across all cycles, the performance of C2 can be considered superior as EPEF, BW, and ADG of C2 are observed to be higher than C0 and C1 (ANOVA, *p* < 0.05, Table 2). However, C2 also demonstrated the highest values in HB scores and prevalence (ANOVA, *p* < 0.05, Table 2). From this, T1 and T2 were observed to have the lowest HB metrics, while T4 exhibited the highest (ANOVA, *p* < 0.05, Table 1).

**Table 1:** Performance and health parameters of birds across treatment groups grouped according to cycles

	**T1**	**T2**	**T3**	**T4**	**T5**	**T6**	**SEM**	**MSE**	**F-stat**	***p*-val**
C0										
BW	2.435	2.405	2.436	2.397	2.383	2.376	0.011	0.005	0.819	0.546
FCR	1.368b	1.393ab	1.389ab	1.418a	1.410a	1.412a	0.005	0.001	2.891	**0.030**
ADG	60.876	60.118	60.902	59.933	59.580	59.410	0.282	2.949	0.819	0.546
MT	3.500	2.867	3.133	3.167	3.033	3.167	1.249	1.249	0.209	0.956
EPEF	406.007	398.163	402.015	389.479	390.510	388.676	2.662	244.196	1.312	0.285
FPDS	0.027	0.020	0.027	0.010	0.007	0.010	0.003	0.000	1.440	0.239
FPDP	2.667	2.000	2.667	1.000	0.667	1.000	0.314	3.333	1.440	0.239
HBS	0.010	0.013	0.007	0.000	0.007	0.003	0.002	0.000	1.200	0.333
HBP	1.000	1.333	0.667	0.000	0.667	0.333	0.178	1.111	1.200	0.333
C1										
BW	2.417	2.418	2.439	2.412	2.378	2.436	0.0101	0.004	0.731	0.606
FCR	1.425	1.426	1.415	1.447	1.436	1.426	0.004	0.001	1.146	0.358
ADG	58.945	58.967	59.480	58.832	58.009	59.419	0.248	2.300	0.731	0.606
MT	1.767	2.200	2.600	2.867	1.833	2.433	0.128	0.501	2.250	0.075
EPEF	383.464	383.409	386.183	371.883	377.150	381.341	2.150	167.042	0.975	0.449
FPDS	0.020	0.010	0.043	0.020	0.017	0.027	0.004	0.001	0.516	0.762
FPDP	1.667	1.000	3.333	2.000	1.333	2.000	0.308	3.556	0.725	0.610
HBS	0.030	0.027	0.033	0.037	0.023	0.023	0.005	0.001	0.973	0.450
HBP	2.000	2.000	2.667	2.667	2.000	1.667	0.312	3.533	0.962	0.456
C2										
BW	2.648	2.625	2.629	2.603	2.586	2.597	0.013	0.006	0.540	0.744
FCR	1.398d	1.428bc	1.417c	1.459a	1.442a	1.442ab	0.004	0.000	17.473	** < 0.001**
ADG	64.591	64.026	64.120	63.477	63.062	63.338	0.307	3.633	0.540	0.744
MT	2.000	2.567	2.900	2.033	2.800	2.367	1.011	0.166	0.857	0.521
EPEF	429.986a	412.178bc	416.671ab	401.411c	399.630c	403.987bc	2.557	141.778	5.617	**<0.001**
FPDS	0.230	0.243	0.213	0.260	0.263	0.260	0.012	0.006	0.401	0.844
FPDP	17.333	17.667	16.667	19.333	19.333	19.000	0.945	36.222	0.218	0.952
HBS	0.153b	0.153b	0.200ab	0.243a	0.190ab	0.210ab	0.009	0.002	3.214	**0.019**
HBP	13.000b	12.000b	15.333ab	17.667a	14.000ab	15.667ab	0.562	9.133	2.727	**0.038**

Cycle 0 (C0), Cycle 1 (C1), and Cycle 2 (C2) (*N* = 108 pens, 36 pens/cycle). Performance parameters taken as mean per pen: Bird weight (*BW*) - in kilograms, average daily gain (*ADG*) — grams per day, feed conversion ratio (*FCR*), total mortality (*MT*) - %, European production efficiency factor (*EPEF*), footpad dermatitis score (*FPDS*) and prevalence (*FPDP*) - %, and hock burn score (*HBS*) and prevalence (*HBP*) - %. Significant differences between treatment groups (ANOVA, *p* < 0.05). Different letters denote significant differences using DMRT grouping (*p* < 0.05). *SEM* standard error of the mean, *MSE* error mean sum of squares, *F-stat* (ANOVA F-statistic)

**Table 2:** Performance and health parameters of birds across cycles

	**C0**	**C1**	**C2**	**MSE**	**F-stat**	***p*-val**
BW	2.405b ± 0.011	2.417b ± 0.01	2.615a ± 0.013	0.005	106.555	< 0.001
FCR	1.399b ± 0.005	1.429a ± 0.004	1.390b ± 0.005	0.001	19.345	< 0.001
ADG	60.136b ± 0.282	58.942c ± 0.248	63.769a ± 0.307	2.826	80.499	< 0.001
MT	3.144a ± 0.175	2.283b ± 0.128	2.444b ± 0.166	0.896	8.420	< 0.001
EPEF	395.808b ± 2.662	380.572c ± 2.15	410.644a ± 2.557	218.944	37.176	< 0.001
FPDS	0.017b ± 0.003	0.023b ± 0.004	0.245a ± 0.012	0.002	282.571	< 0.001
FPDP	1.667b ± 0.314	1.889b ± 0.308	18.222a ± 0.945	13.046	248.782	< 0.001
HBS	0.007c ± 0.002	0.029b ± 0.005	0.192a ± 0.009	0.001	283.873	< 0.001
HBP	0.667c ± 0.178	2.167b ± 0.312	14.611a ± 0.562	5.348	394.411	< 0.001

Cycle 0 (C0), Cycle 1 (C1), and Cycle 2 (C2) (*N* = 108 pens, 36 pens/cycle). Performance parameters taken as mean per pen: Bird weight (*BW*) - in kilograms, average daily gain (*ADG*) — grams per day, feed conversion ratio (*FCR*), total mortality (*MT*) - %, European production efficiency factor (*EPEF*), footpad dermatitis score (*FPDS*) and prevalence (*FPDP*) - %, and hock burn score (*HBS*) and prevalence (*HBP*) - %. Significant differences between treatment groups (ANOVA, *p* < 0.05). Different letters denote significant differences using DMRT grouping (*p* < 0.05). *MSE*, error mean sum of squares; *F-stat* (ANOVA F-statistic)

To evaluate the overall effects of the individual GHI components across the three cycles, we employed LASSO regression. As shown in Table 3, all GHI components were revealed to be significant predictors of at least one of the performance parameters. Notably, EPEF was largely influenced by all variables, while MT was not associated with any. Analysis showed that ionophore administration at all stages can largely increase EPEF but slightly decrease FCR. Meanwhile, the opposite was demonstrated by the administration of ionophores only during the finisher stage. Vaccination was shown to have only a slight negative effect on EPEF, while the use of probiotic A was shown to negatively affect almost all parameters. In contrast, the use of probiotic B was generally associated with positive changes with respect to all parameters, apart from FCR and MT. The remaining predictors were shown to have large negative impacts on EPEF but a very low positive impact on FCR.

**Table 3:** LASSO regression of performance and health parameters of broilers across all cycles

Predictors	Performance parameters						
	**ADG**	**BW**	**EPEF**	**FCR**	**FPDP**	**HBP**	**MT**
(Intercept)	61.207	2.489	397.937	1.400	7.354	5.294	2.624
Ionophore (All stages): yes	0.000	0.000	8.517	− 0.019	0.000	0.000	0.000
Ionophore (Finisher only): yes	0.000	0.000	− 10.152	0.019	0.000	0.000	0.000
Vaccinated: Yes	0.000	0.000	− 0.002	0.000	0.000	0.000	0.000
Probiotic A: Yes	− 1.438	− 0.069	− 3.313	0.000	− 5.481	− 3.651	0.000
Probiotic B: Yes	1.931	0.099	17.376	− 0.018	10.534	9.644	0.000
1% reduced crude protein: Yes	0.000	0.000	− 13.893	0.034	0.000	0.000	0.000
Essential oil: Yes	− 0.427	− 0.017	− 10.748	0.022	0.000	0.000	0.000
Prebiotic: Yes	0.000	0.000	− 15.617	0.024	0.000	0.000	0.000

LASSO regression against sources of variability where the GHI individual components are coded as 1/0, corresponding to “yes”/ “no”. The model assumes an L1 penalty term, which forces the beta coefficient of insignificant predictors to become zero. Significant negative and positive beta coefficients indicate a decrease and increase in performance parameters, respectively. Average daily gain (*ADG*) — grams per day, bird weight (*BW*) - in kilogram, European production efficiency factor (*EPEF*), feed conversion ratio (*FCR*), footpad dermatitis prevalence (*FPDP*) - %, hock burn prevalence (*HBP*) - %, mortality (*MT*) - %

### Administration of different GHIs resulted in shifts in microbial community structure and diversity

**Fig. 2:**
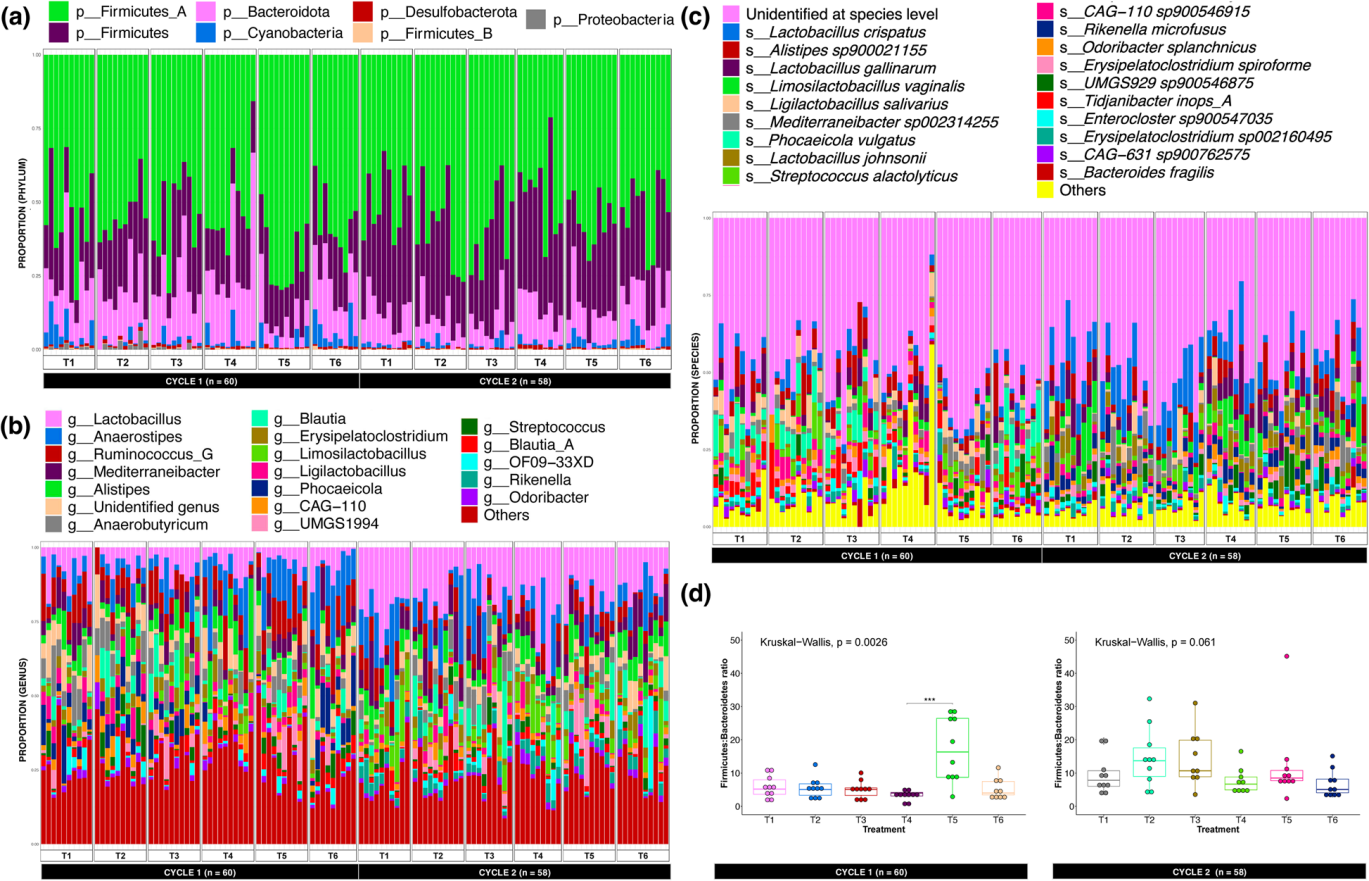
Sample-wise distribution of 84 MAGs recovered from C1 (*n* = 60) and C2 (*n* = 58). Proportions at **a** phylum, **b** genus (genera with < 2% prevalence grouped into “Others”), and **c** species levels (species with < 1% prevalence grouped into “Others”), ranked from most dominant to least and grouped per treatment/cycle. The plot also shows **d** Firmicutes-Bacteroidota ratio, grouped per cycle and treatment, with significant differences based on Kruskal–Wallis (*p* < 0.05) and pairwise Dunn testing with Bonferroni correction (*p* < 0.001)

**Fig. 3:**
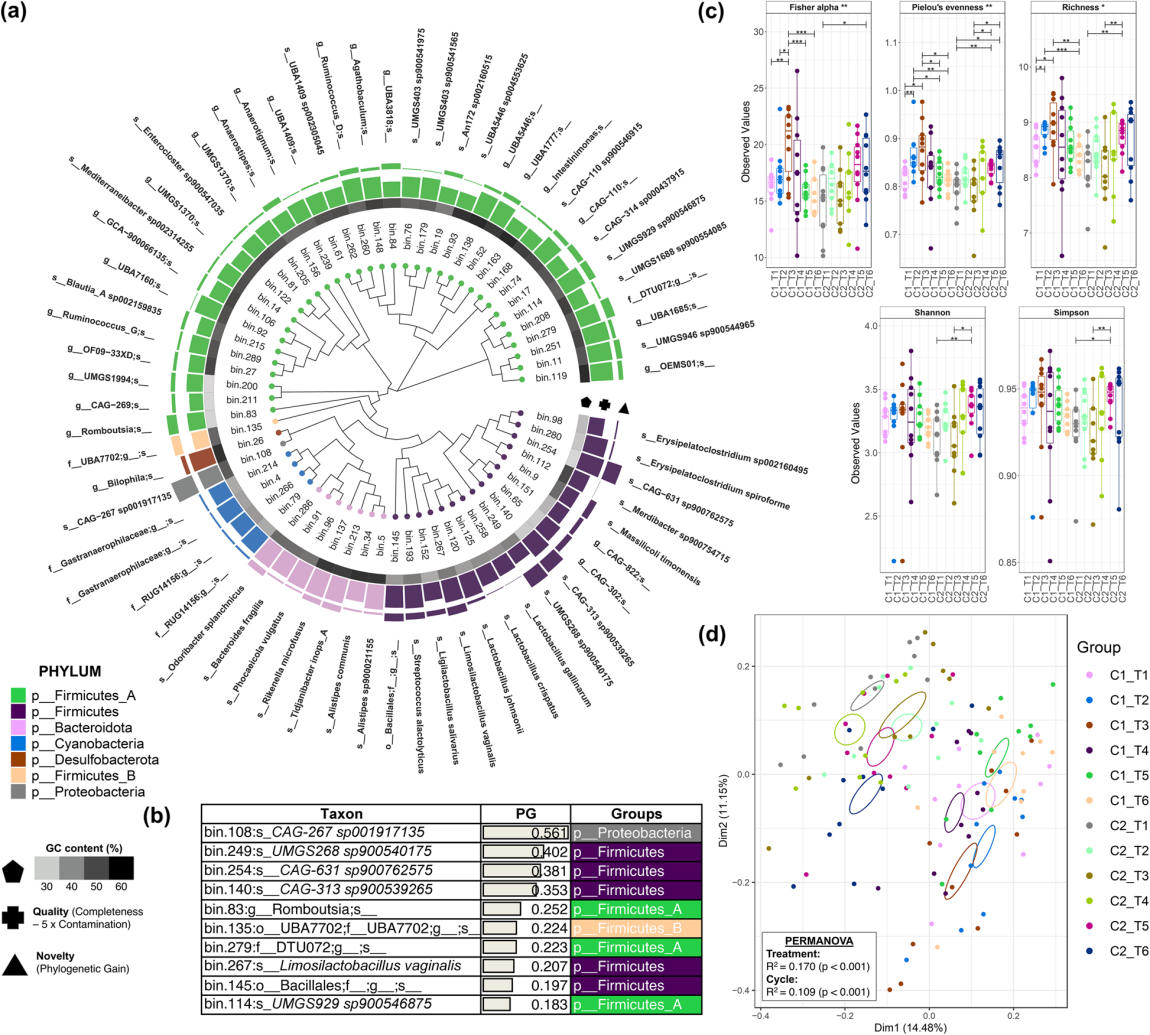
Phylogeny and taxonomic diversity of MAGs recovered from C1 (*n* = 60) and C2 (*n* = 58). **a** A phylogenetic tree of 65 MAGs recovered via GToTree using 25 bacterial and archaeal specific single copy genes. The tree also features G-C content, quality index (genome completion — 5 × genome contamination), and novelty (represented by phylogenetic gain (PG) values calculated using the GTDB-Toolkit). **b** Ten most novel MAGs (indicated by high PG). Finally, alpha diversity (**c**) is represented by Fisher alpha, Pielou’s evenness, rarefied richness, and Shannon and Simpson index, with ANOVA significance: *p* < 0.001 (***), *p* < 0.01 (**), *p* < 0.05 (*). **d** Beta diversity is represented by PCoA plot of Bray–Curtis indices, with PERMANOVA (*p* < 0.001)

The sequencing analysis of 118 caecal samples yielded a total of 2.6 B reads, from which a total of 84 MAGs with greater than 75% completeness and less than 5% contamination were recovered. Specifically, 83 of these MAGs are detected in C1, while 78 are present in C2. All included MAGs were identified as bacterial species, which represent seven unique phyla, with Firmicutes_A as the most common designated phylum among all samples (Figs. 2 and 3, Supplement File 2). This was followed by Firmicutes and Bacteroidota, while MAGs belonging to Proteobacteria were not present in any of the groups in C2 (Fig. 2a). The global microbiota abundance was dominated by MAGs distributed across 57 identified genera, accounting for 96% of the population. Among these, we identified *Lactobacillus* as the predominant genus, while its member, *Lactobacillus crispatus*, was the most common species. However, predominant MAGs at genus and species levels were observed to vary largely across treatments and cycles (Fig. 2, Supplement File 2). For instance, *Ruminococcus_G* was observed to have the highest proportion in T1 and T3; *Anaerobutyricum* in T2, T5, and T6; and *Alistipes* for T6, while *Lactobacillus* was consistently highest in proportion among C2 treatment groups. We also assessed Firmicutes-Bacteroidetes ratio of each treatment group, where we considered Firmicutes as the sum of Firmicutes, Firmicutes_A, and Firmicutes_B counts. Comparative analysis of the F/B ratio revealed significant differences between treatment groups in C1 only, wherein T5 is observed to have a higher ratio than T4 (Fig. 2d). Approximately, 77% of the MAGs (*n* = 65) contained SCGs for phylogenetic mapping, showing four major groupings which were dominated by Firmicutes_A species (Fig. 3a). Among these MAGs, bin.108 (*CAG-267 sp001917135*) was revealed as the most novel species (Fig. 3b).

Next, we hypothesised that GHI groups may influence microbial community diversity, which we then estimated using various alpha- and beta-diversity metrics. In C1, microbial communities of T3 samples were found to be richer and possessed a more even distribution than other groups (T1, T2, T4, and T6), as indicated by significantly higher FA, R, and PE values. Meanwhile, in C2, the majority of alpha-diversity metrics of T5 and T6 were significantly elevated compared to T1 and T3 (Fig. 3c). The principal components (PC1 and PC2) of our PCoA plot on Bray–Curtis estimates explained a considerable portion of the variance (14.5% and 11.1%, respectively), from which a discernible separation (PERMANOVA, *p* < 0.001, *R*^2^ = 10.9%) between C1 and C2 groups was observed. While separation between treatment groups is not distinct, significant differences between all treatment groups were identified (PERMANOVA, *p* < 0.001, *R*^2^ = 17%). Clustering reveals a slight separation of T2 and T3 (in C1) from the other groups, while T1, T4, and T6 (in C2) are separated from the rest (Fig. 3d).

### Metagenomic-assembled genome composition is associated with the administration of different GHIs and broiler performance

**Fig. 4:**
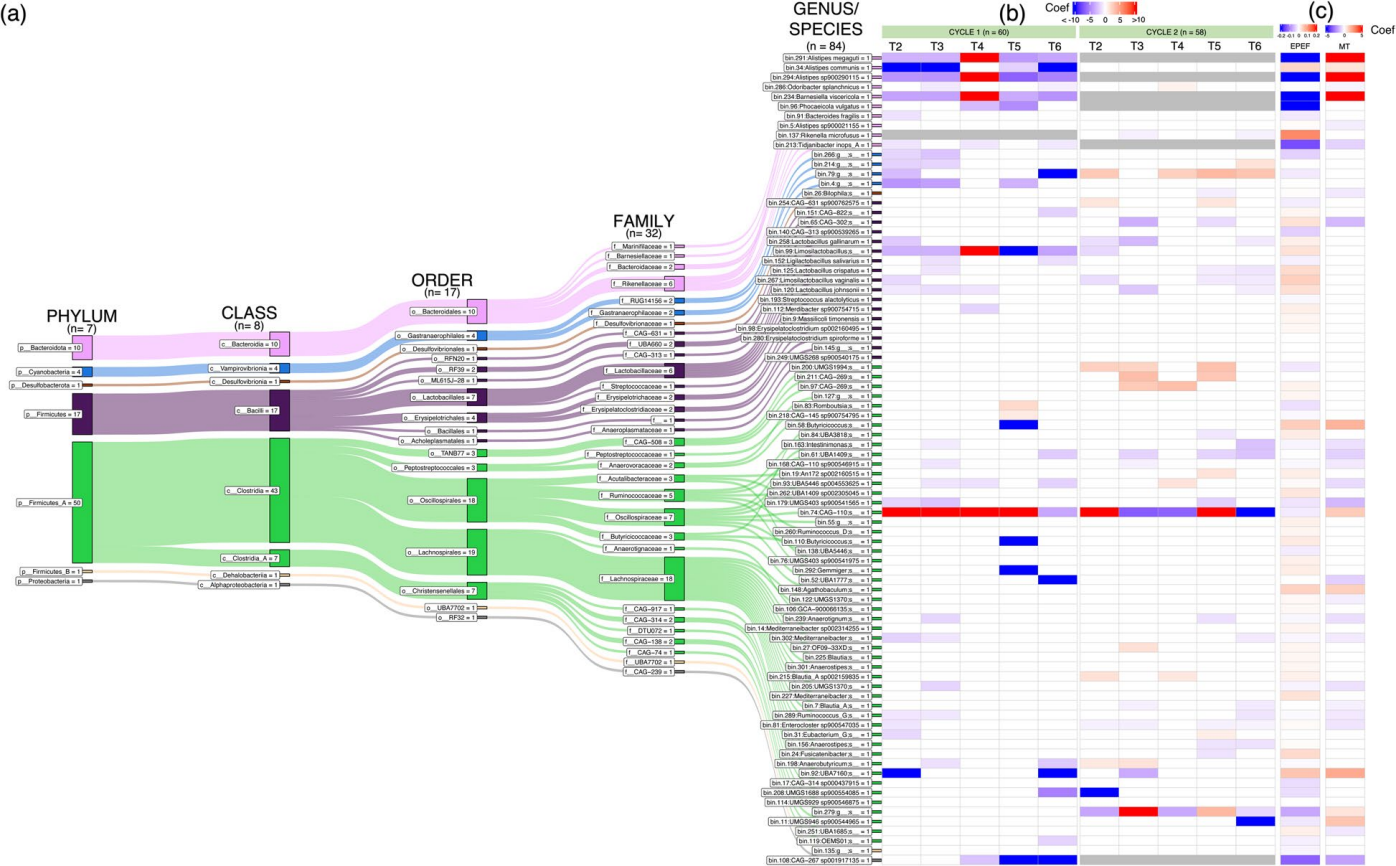
Taxonomic classification and differential analysis of 84 MAGs recovered from C1 (*n* = 60) and C2 (*n* = 58). **a** A Sankey plot illustrating the classification of the bacterial species at various taxonomic ranks. The figure also includes general linear latent variable model (GLLVM) results showing the association of MAGs with **b** treatment groups (with C1 and C2 done separately) and **c** performance and health parameters — EPEF (European performance efficiency factor) and MT (total mortality). Coef, GLLVM coefficients in blue (< 0) or red (> 0), representing negative or positive coefficients (or decrease or increase) for each MAG against GHI treatments (T2, T3, T4, T5, T6) as predictors in comparison to T1 (reference). Coefficients in white indicate insignificance (no association), while grey indicates that the MAG was not recovered in that treatment group

To further understand the individual influence of GHI groups on microbiome and performance, we conducted a GLLVM regression analysis of each of the 84 MAGs (using T1 as the reference predictor). A total of 43 and 38 MAGs (59 for both) were observed to have a significant association with GHI groups in C1 and C2, respectively (Fig. 4, Supplement File 1). For C1, the majority of these MAGs (*n* = 41/43, 95%) exhibited a negative association with GHI administration, denoting a decrease in abundance compared to T1. From this, T3 (21/43, 49%) had the greatest number of decreased MAGs, followed by T2 (20/43, 47%) and T6 (19/43, 44%). Meanwhile, 7 (7/43, 16%) MAGs were observed to be positively associated with GHI administration in C1, from which 5 MAGs (5/43, 12%) increased in T4. Notable MAGs to consistently change across groups in C1 were those belonging to Bacteroidales (bin.291 *Alistipes megaguti*, bin.34 *Alistipes communis*, bin.294 *Alistipes* *sp900290115*, bin.234 *Barnesiella viscericola*), as well as bin.99 (*Limosilactobacillus*) and bin.74 (CAG-110). Bin.34 (*A. communis*) was also observed to have the lowest GLLVM coefficient, followed by bin.92 (g_*UBA7160*) and bin.4 (f_*Gastrophilaceae*). In addition, all Cyanobacteria MAGs were observed to show a negative association with GHI groups.

Among the 38 MAGs identified as associated with C2 groups, 15 (39%) were positively associated with MAGs, while 25 (66%) MAGs were negatively associated. The T5 group had the highest count (*n* = 8/38, 21%) of positively associated MAGs, while T6 had the highest number of negatively associated MAGs (*n* = 15/38, 39%). Bin.74 (*g_CAG-110*) was observed to be dynamic across all GHI groups, as well as having the highest coefficient among all MAGs in C2, while bin.208 (*UMGS1688 sp90054085*) was shown to have the strongest negative association (Fig. 4b).

For performance, we considered metrics EPEF and MT to represent overall broiler health and performance (EPEF was highly correlated with the other measured performance parameters), as shown in Fig. 4c. Our analysis revealed that EPEF was positively associated with 40 MAGs and negatively associated with 33 MAGs, while MT was associated with only 30 MAGs (10-positive and 20-negative associated MAGs). Bin.137 *(Rikenella microfusus*) was noted to have the highest GLLVM coefficient against EPEF, followed by bin.92 *(g_UBA7160*) and bin.58 (*Butyricicoccus*). Bin.234 (*B.* *viscericola*) was observed to have the strongest negative association with EPEF but also the strongest positive association with MT (Fig. 4c, Supplement file 1). Interestingly, at least one of the Lactobacillaceae MAGs (bin.258 *Lactobacillus gallinarum*, bin.99 *Limosilactobacillus*, bin.152 *Ligilactobacillus*, bin.125 *L. crispatus*, bin.267 *Limosilactobacillus salivarius*, bin.120 *Lactobacillus johnsonii*) were negatively associated across the GHI groups in both cycles (except for T4) but were also seen to have positive association with EPEF. This finding indicates that increased levels of Lactobacillaceae MAGs in GHIs may contribute to greater EPEF values, but they were reduced in the GHI groups compared to T1. Similar patterns were also seen for other MAGs, such as bin.34 (*A. communis*), bin.137 (*R. microfusus*), bin.65 (*CAG-302*;s__), and bin.84 (*UBA3818*;s__). Furthermore, numerous MAGs enriched in GHI groups but negatively associated with EPEF were also identified (Cyanobacteria MAGs, bin.200: *UMGS1993*;s__, bin.279: f_DTU072;g__;s__ in C2) which signifies that elevated levels of these MAGs can contribute to decreased levels of EPEF.

### Administration of different GHIs resulted in shifts in metabolic functions

**Fig. 5:**
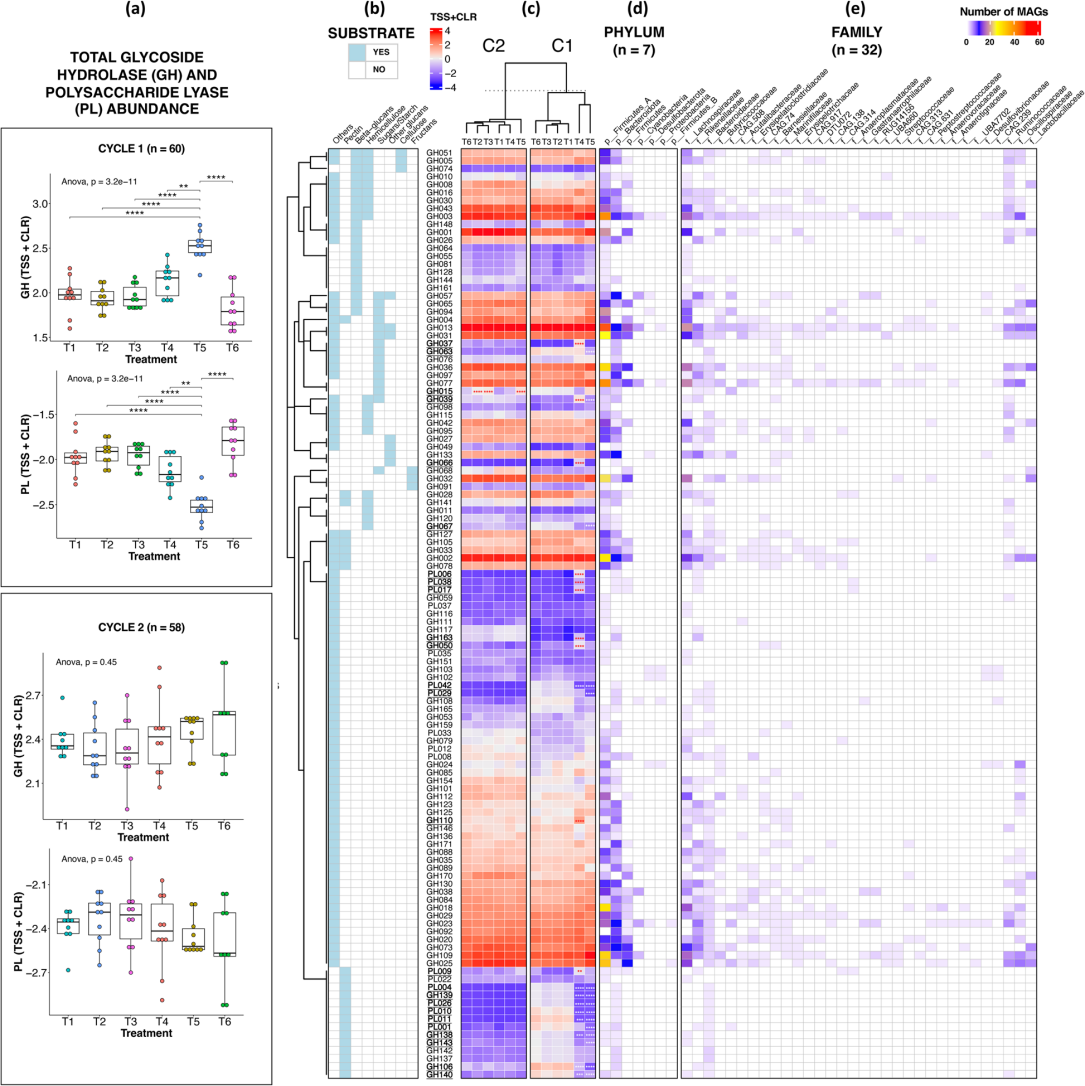
Carbohydrate-active enzymes (CAZyme) gene abundance recovered from C1 (*n* = 60) and C2 (*n* = 58). **a** Comparison of total glycoside hydrolase (GH) and polysaccharide lyase (PL) abundances across treatment groups and cycles, based on ANOVA and post hoc pairwise *t*-testing with Bonferroni correction. **b** CAZyme IDs grouped according to substrate based on the dbCAN2 and MetaCyc databases). **c** Mean normalised abundance of CAZyme gene IDs across treatment groups and cycles. Red and blue colour of heatmap cells indicates high and low abundance, respectively. IDs in bold and underlined indicate significant increase or decrease in abundance compared to T1 based on Wald test (*DESeq2*). Number of MAGs containing CAZyme genes, grouped according to **d** phylum and **e** family taxonomic ranks. Normalisation method: total sum scaling and centralised log ratio (TSS + CLR). Significance: *p* < 0.0001 (****), *p* < 0.001 (***), *p* < 0.01 (**), *p* < 0.05 (*). White and red significance indicate a decrease and increase of MAGs in treatments T2 to T6 compared to T1, respectively

Since significant differences in MAGs were observed across treatments, we then investigated the impact of GHIs on metabolic functions. Genes encoding for enzymes, including CAZymes and proteases, are important for the metabolism and reproduction of microbial species. Hence, they may also play crucial roles in the nutrition and digestive physiology of chickens. As seen in Fig. 4, we detected a total of 128 CAZymes belonging to two major families — glycoside hydrolase (GH) and polysaccharide lyase (PL), with the former being more dominant. Both CAZyme families were significantly different in abundance across C1 treatment groups, revealing T5 to have the highest GH abundance but also the lowest PL abundance (*p* < 0.05, ANOVA) (Fig. 5a). Meanwhile, there were no significant differences between groups within C2, but they have a relatively higher overall CAZyme abundance than C1 groups. Furthermore, clustering analysis exhibited the separation of T4 and T5 from the other groups in C1 and T5 in C2 (Fig. 5c).

The most common enzyme across all treatments was observed to be GH013, which was detected in 27 bacterial families in our study. A total of 24 enzymes were noted to have significantly different abundances between T1 and GHI groups T4 and T5 (C1) (Wald test, *p* < 0.05, Fig. 5c). For T4, 8 enzymes with activity against pectin were significantly lower than T1, while 9 enzymes with activity against starch/sugars and other carbohydrates were significantly higher. Similarly, 15 enzymes with activity against pectin and hemicellulose are downregulated in T5; these same enzymes were observed to be consistently present in the family Bacteroidaceae (Fig. 5e). In C2, only GH15 was observed as significantly differentiated (upregulated in T2, T3, and T5), which has the capacity for sugars/starch digestion.

**Fig. 6:**
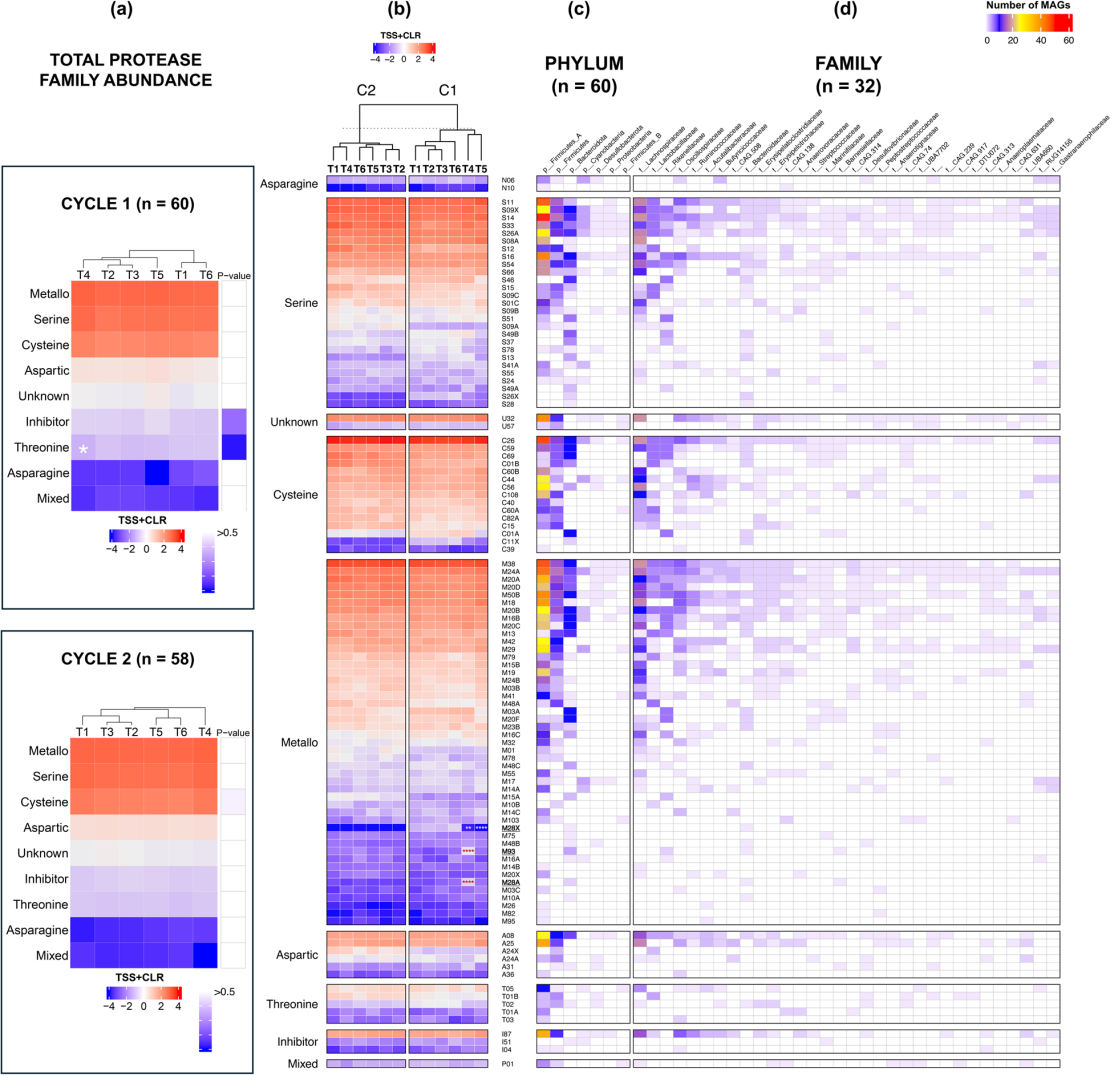
Mean normalised abundance of proteases across treatment groups and cycles. **a** Mean abundance of individual protease families (MEROPS ID) grouped according to protease type following MEROPS database [49]. MEROPS protease ID in bold and underlined indicates a significant difference in abundance compared to T1, based on the Wald test (using *DESeq2*). **b** Mean abundance of total protease grouped according to family across groups and cycles, and statistical differences were based on Kruskal-Wallis test and post hoc pairwise Dunn testing with Bonferroni correction. Number of MAGs with protease gene grouped according to taxonomic ranks **c** phylum and **d** family. Normalisation method: total sum scaling and centralised log ratio (TSS + CLR). Significance: *p* < 0.0001 (****), *p* < 0.001 (***), *p* < 0.01 (**), *p* < 0.05 (*), white and red significance indicates decrease and increase of MAGs in GHI treatments (T2, T3, T4, T5, T6) compared to T1, respectively

The differential abundance of proteases across treatments was also investigated in this study. A total of 108 protease families distributed across 9 protease types were detected in our samples (Fig. 6). Metallo peptidases were detected as the most common and most diverse protease catalytic type, with the M38 family being the most dominant across 47 metallo families. Other detected catalytic types included inhibitors and threonine peptidases, which were both significantly different in abundance in C1, where threonine abundance was specifically observed to be significantly lower in T4 compared to T1 (Dunn test with Bonferroni correction, *p* < 0.05). In C2, abundances of cysteine peptidases were markedly disparate (Kruskal-Wallis test, *p* < 0.05), with T4 having the highest value among all groups but although insignificant. At the family level, divergent clustering of T4 and T5 from other groups was seen in C1; in contrast, for C2, T1 and T4 were seen to be more similar in abundance than the other groups. Across all treatment groups, C38 (cysteine) was consistently the most abundant peptidase, followed by M38 and S33. However, diminished levels of M28X were evident in T4 and T5, while elevated levels of M93 and M28A were observed in T4 in C1. Notably, all these differentiated families were present in the Bacteroidaceae family (Fig. 6d).

**Fig. 7:**
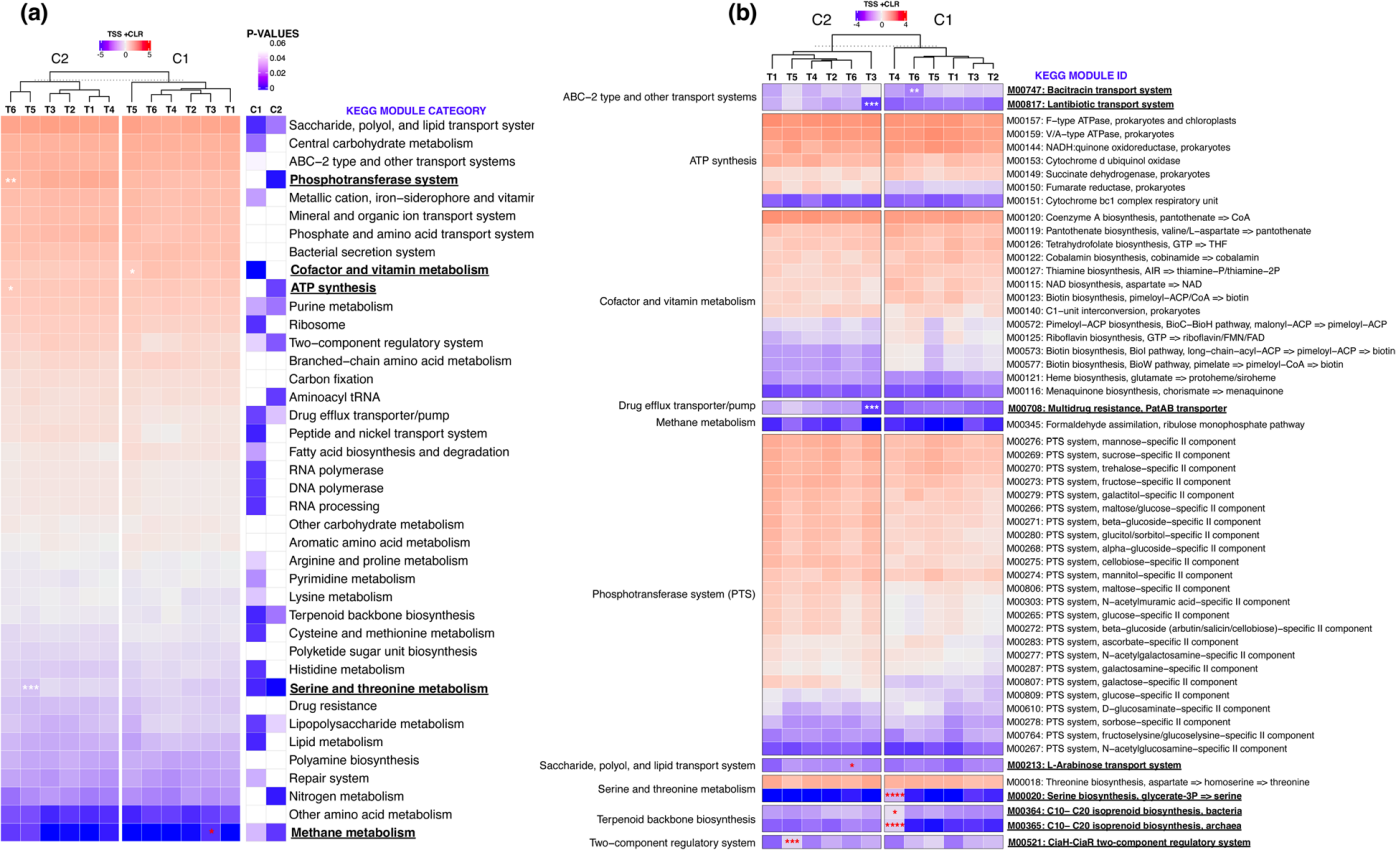
KEGG metabolic module abundances across treatment and cycles. Mean normalised abundance of **a** KEGG module categories and respective *p*-values based on Kruskal-Wallis testing and **b** selected KEGG modules across treatment groups and cycles (C1 and C2). The red and blue colour of heatmap cells indicates high and low abundance, respectively. KEGG category and module IDs in bold and underlined indicate significant differences in abundance compared to T1 based on post hoc pairwise Dunn testing with Bonferroni correction and *DESeq2*, respectively. Normalisation method: total sum scaling and centralised log ratio (TSS + CLR). Significance: *p* < 0.0001 (****), *p* < 0.001 (***), *p* < 0.01 (**), *p* < 0.05 (*). Significance in white and red indicates a decrease and increase of MAGs in GHI treatments (T2, T3, T4, T5, T6) compared to T1, respectively

Gut microorganisms engage in complex metabolic interactions, potentially involving various production of metabolites that modulate host physiology. Based on our analysis, we determined that GHI administration also had an impact on other KEGG modules, including various transport and transporter systems, amino acid metabolism, and nucleic acid metabolism pathways (Fig. 7). Significantly varied abundances across treatment groups in 25 and 12 different KEGG function module categories were observed in C1 and C2, respectively (*p* < 0.05, Kruskal-Wallis test) (Fig. 7a). Heatmap clustering also exhibited the divergence of T5 from the other groups in C1, while both T5 and T6 have partitioned from the others in C2. However, compared to T1, abundance in GHI groups was significantly downregulated in four module categories, namely phosphotransferase system (PTS) and ATP synthesis in C2–T6, serine and threonine metabolism in C2–T5, and co-factor and vitamin metabolism in C1–T5, while methane metabolism function is upregulated in C1–T3. Furthermore, differential analysis (T1 as reference) of individual modules revealed a decreased abundance of antibiotic-related transport systems in GHI groups C1–T6 (M00747) and C2–T3 (M00817, M00708) and an increase of organic compound biosynthesis and transport and regulatory system modules in C1–T4 (M00020, M00364, M00365), C2–T6 (M00213), and C2–T5 (M00521) (Fig. 7b).

### Administration of different GHIs can impact short-chain fatty acid production pathways

**Fig. 8:**
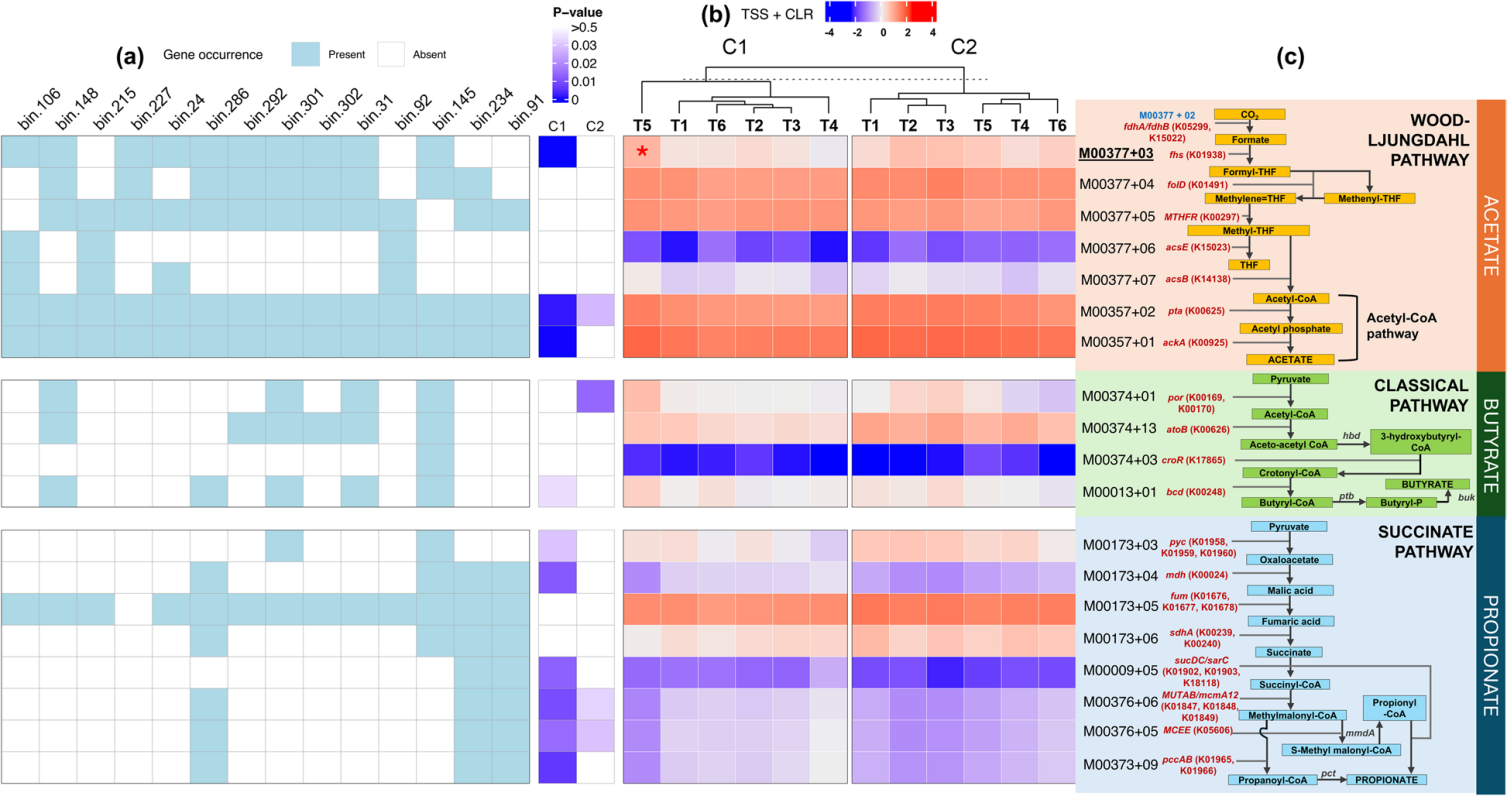
Metagenomic abundance of elements of short-chain fatty acids (SCFA) pathways. **a** Selected MAGs containing the most complete SCFA submodules. Blue and white colour indicates the presence and absence of element, respectively. **b** Heatmap of normalised abundances of SCFA enzyme corresponding module IDs across treatment groups and cycles (C1 and C2), with significance based on Kruskal-Wallis test (*p* < 0.05 in blue). TSS + CLR, total sum scaling and centralised log ratio. The red and blue colour of heatmap cells indicates high and low abundance, respectively. **c** SCFA pathways showing KEGG orthology (KO) codes and enzyme names (in red). Submodule and significance in red: significant increase based on pairwise Dunn test with Bonferroni correction (*p* < 0.05). Submodules in blue: not detected in the metagenome dataset in this study. Enzyme names in black: no matching submodule ID in the KEGG database [46]. Enzymes are listed as follows: *fdhA*, formate dehydrogenase alpha subunit; *fdhB*, formate dehydrogenase beta subunit; *fhs*, formate tetrahydrofolate ligase; *foID*, methylenetetrahydrofolate dehydrogenase; *MTHFR*, methylenetetrahydrofolate reductase; *acsE*, 5-methyltetrahydrofolate corrinoid/iron sulphur protein methyltransferase; *acsB*, acetyl-CoA synthase; *pta*, phosphate acetyltransferase; *ackA*, acetate kinase; *por*, pyruvate ferredoxin oxidoreductase; *atoB*, acetyl-CoA C-acetyltransferase; *hbd*, 3-hydroxybutyryl-CoA dehydrogenase; *croR*, 3-hydroxybutyryl-CoA dehydratase; *bcd*, butyryl-CoA dehydrogenase; *ptb*, phosphate butyryltransferase; *pyc*, pyruvate carboxylase; *buk*, butyrate kinase; *mdh*, malate dehydrogenase; *fum*, fumarate hydratase; *sdhA*, succinate dehydrogenase; *sucD*, succinyl-CoA synthetase alpha subunit; *sucD*, succinyl-CoA synthetase beta subunit; *aarC*, succinyl-CoA:acetate CoA-transferase; *MUTAB*, methylmalonyl-CoA mutase alpha and beta; *mcmA1*, methylmalonyl-CoA mutase, N-terminal domain; *mcmA2*, methylmalonyl-CoA mutase, C-terminal domain; *mmdA*, methylmalonyl-CoA decarboxylase subunit alpha; *MCEE* epi, methylmalonyl-CoA/ethylmalonyl-CoA epimerase; *pccA*, propionyl-CoA carboxylase alpha chain; *pccB*, propionyl-CoA carboxylase beta chain; *pct*, propionate CoA-transferase

Due to the known relevance of metabolic pathways involved in the production of short-fatty acids (SCFA), including acetate, butyrate, and propionate, on gut health, we also explored the effects of GHI on SCFA production. As shown in Fig. 8, the majority of KEGG submodules related to SCFA production were present in our samples (excluding M00377 + 02). We also identified MAGs to have complete sets of modules for the acetyl-CoA pathway (48 out of 84 MAGs) and butyrate pathways (bin.145: Bacillales;f__;g__;s__). However, none of our MAGs possessed complete sets of submodules for Wood-Ljungdahl and propionate pathways. Bin.92 (*UBA7160*;s__) and bin.215 *(Blautia_A sp002159835*) have the highest number of submodules (*n* = 5) for acetate production, while the MAGs bin.91 (*Bacteroides fragilis*) and bin.234 (*B. viscericola*) have all of the propionate-related submodules except for M00173 + 03 — a submodule for pyruvate carboxylase (*pyc*) (Fig. 8a). Across our treatment groups, 11 out of the included 19 SCFA submodules were significantly varied, wherein propionate submodules were observed as mostly affected. From this, post hoc pairwise comparison revealed a single elevation of M00377 + 03 in C1–T5 (Fig. 8b). Furthermore, distinct separation of T5 in C1 and distancing of T1 and T6 from other groups in C2 were observed.

### MAGs associated with better performance have a higher capacity for nutrient digestion and metabolism

**Fig. 9:**
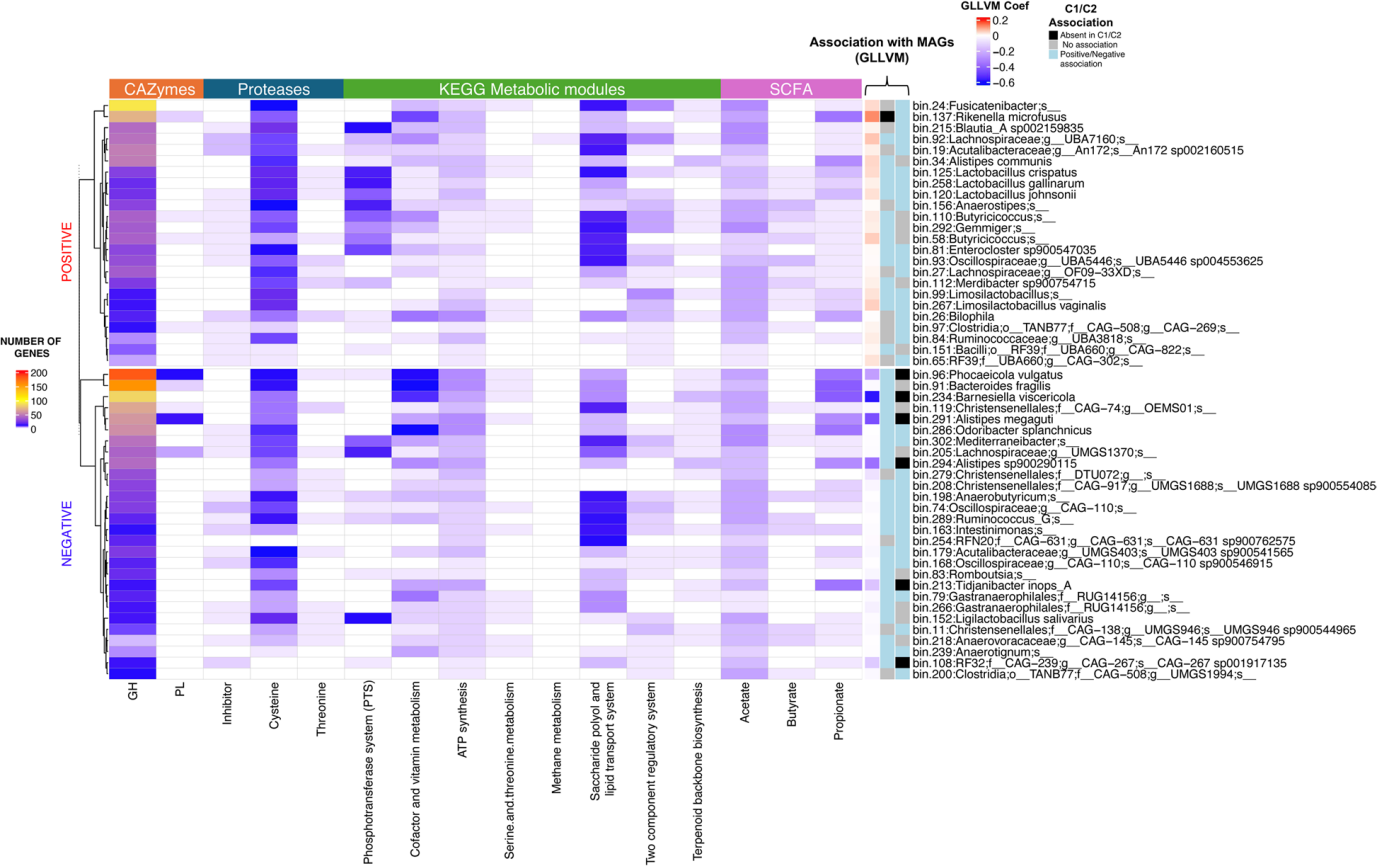
Overview of significant metagenomic features of 52 MAGs associated with EPEF and GHIs. EPEF, European poultry efficiency factor; GH, glycoside hydrolase; GHI, gut health interventions; GLLVM, generalised latent linear variable model; MAG, metagenome-assembled genomes; SCFA, short-chain fatty acids; PL, polysaccharide lyase

Based on the above analysis, we explored the gene abundance of several metagenomic features (which were selected based on statistical significance among treatment groups) of 52 MAGs that were revealed to be associated with both EPEF and composition in GHI groups (Fig. 9). Clustering analysis showed that MAGs with a positive association with EPEF (EPEF +) have an overall higher number of genes encoding for the various significant metabolic features. Specifically, the EPEF + group demonstrated higher genes in the PTS module, butyrate and propionate SCFA module, while similar abundance in CAZymes and proteases between the two EPEF groups can be seen. However, it is interesting to point out that *R. microfusus*, the MAG with the highest GLLVM coefficient for EPEF, do not possess any genes for PTS and butyrate production modules.

## Discussion

The microbial community structure of the gut has been proven crucial in both host health and performance [1, 8]. As such, supplementation of feed additives to diets and administration of other health interventions have become common approaches to modulate intestinal microbial communities and ensure the ideal growth and health of broilers [71]. Previously, we reported alterations in the chicken gut microbiome in response to various production systems using 16S rRNA sequencing. Our findings revealed that the inclusion of omega-3 in feed can result in an increase in bacterial genera associated with short-chain fatty acid (SCFA) production and affect levels of pathogenic bacteria, including *Campylobacter* levels, through competitive exclusion [9]. Due to the increasing demand for poultry meat worldwide [72], there is a need to optimise gut health for improved feed efficiency and overall health of broilers. Therefore, we designed modified gut health schemes across three broiler production cycles and assessed their influence on the caecal microbiome using metagenomic shotgun sequencing. Herein, we employed the assembly of 84 high-quality MAGs recovered from 118 caecal samples and analysis of the metabolic function capacity of the MAGs and association with performance parameters and health.

The administration of ionophores and GHIs in this study, as reported in previous research, can significantly impact performance through gut modulation in broilers, though the specific effects may vary across studies [73, 74]. For instance, Zhang et al. [75] reported a significant increase in EPEF, ADG, and BW and a decrease in FCR in birds administered with *Bacillus subtilis* probiotics compared to the negative control group. In contrast, other studies involving the use of other *Bacillus*-based probiotics in broilers generally reported insignificant improvement in the same performance metrics [76, 77]. Within our study, we also observed variations across the three cycles, where C2 groups demonstrated the best performance. Since probiotic B was revealed to have the highest positive impact on EPEF, we posit that the efficacy of probiotic B may be a contributing factor to the comparatively superior performance observed in cycle 2 compared to the other cycles provided with probiotic A. These findings further underscore the importance of strain-specific effects and dosage considerations when implementing probiotic interventions in poultry systems, as reported before [78, 79]. However, cycle variations within the same production system have also been reported previously and are hypothesised to occur due to variations in climate, management, and fluctuations in the microbiota of day-old chicks as affected by hatchery-to-farm transfer [80]. Furthermore, some differences in MAG composition between C1 and C2 brought upon by differences in the GHIs used may contribute to the disparities in performance. For instance, *R. microfusus* (bin.137), positively associated with EPEF but absent in C1, has been identified for its potential probiotic effects, attributed to its role in producing short-chain carboxylic acids, which contribute to maintaining cell structure integrity [81]. Conversely, *Alistipes* spp. (bin.291, bin.294) and *Phocaeicola vulgatus* (bin.96), all observed to be negatively associated with EPEF and present in only C1, have also been implicated in human health issues such as cancer, cardiovascular disorders, and inflammatory-related diseases [82, 83]. Another noteworthy MAG is *B. viscericola* (bin.234), previously reported as an efficient coloniser of chicken caeca [84] but here observed as negatively correlated with EPEF but positively correlated with MT and absent in C2.

Comparison of groups has also revealed substantial differences in microbial diversity and composition between GHI groups and the control (T1). Specifically, our analysis revealed that T1 and T3 had better overall performance but exhibited lower alpha diversity than other groups (in C2). According to Coyte et al. [85], high alpha diversity in the gut microbiome tends to destabilise microbiome communities, potentially leading to decreased ecological stability, which is the ability to return to a natural state after a perturbation. Unstable gut microbial communities are then less likely to maintain beneficial symbiotic relationships and may be more susceptible to disturbances or shifts that could impact the host’s health and productivity. With this, the moderate microbial diversity shown in T1 and T3 may indicate a more balanced and stable gut, thereby becoming supportive of optimal performance. Furthermore, these discrepancies can be explained by MAGs in association with performance that were differentiated among groups. MAGs belonging to Lactobacillaceae (bin.258 *L. gallinarum*, bin.99 *Limosilactobacillus*, bin.125 *L. crispatus*, bin.120 *L. johnsonii*), Butyricicoccaceae (bin.58, bin.110 *Butyricicoccus* spp.), Ruminococcaceae (bin.292 *Gemmiger*), and Lachnospiraceae (bin.81 *Enterocloster* sp900547035, bin.24 *Fusicatenibacter*), which are families known for their beneficial roles in gut health such as SCFA production, gut integrity promotion, and protection against pathogens such as *Salmonella* spp. [86–91], were identified as beneficial for performance but were decreased in different GHI groups in comparison to T1. Meanwhile, MAGs including bin.254 CAG-631 sp900762575, bin.200 UMGS1994, bin.83 *Romboutsia*, and bin.74 CAG-110 were enriched in GHI groups but are negatively associated with EPEF. Similarly, an increase in *Romboutsia* has also been noted in broilers given *B. subtilis* and a coccidiosis vaccine [92] and has been reported to also negatively impact the performance of breeder broilers [93].

The standard diet of broilers usually consists of approximately 70% carbohydrates, encompassing starch, oligosaccharides, and non-starch polysaccharides (NSP) like cellulose, hemicellulose, and pectin [94]. These NSPs remain undigested by the host, serving as substrates for the gut microbiome. Consequently, gut microorganisms possess a diverse range of genes encoding enzymes known as CAZymes, which facilitate the breakdown and metabolism of these polysaccharides [95]. CAZymes are categorised into families, including polysaccharide lyases (PL) and glycoside hydrolases (GH), based on sequence similarities, although members within the same family may exhibit different substrate specificities [95]. In our study, we observed a preference of gut microbiota in T4 for “other glucans” and “other glycans” and depletion of pectin bacterial specialists in T4 and T5 of C1. Since the availability of readily accessible growth substrates diminishes as they pass through the gastrointestinal tract [96], we hypothesise that due to the limited availability of protein (amino acids) in T4, more preferred substrates such as starch and pectin have been digested in the upper intestines, leaving caecal microbiota to use other glycans as substrate. Meanwhile, depletion in T5 may be explained by the reduction of Bacteroidia bacteria (in T5), similarly reported by Ding et al. [97], which are microorganisms shown here to digest pectin.

Digestion of protein available in the diet is also of great importance for the optimisation of gut health [98]. However, there is limited information on its association with gut microbial functions in poultry. Previous research has shown that approximately 20% of crude protein (CP) taken in by broilers goes undigested due to insufficient concentrations of endogenous proteinases in the host [96, 99]. Consequently, undigested proteins (or ileal bypass protein), which are fermented by gut microbiota in the hindgut (caeca), can encourage increased growth of *Clostridium perfringens* and production of detrimental metabolites, including ammonia, indoles, and phenols [96]. Hence, we included the reduction of CP as one of our gut health approaches in our study (as represented by T4). Nonetheless, this group was shown to have significantly lower overall performance. Since restriction of CP might have resulted in a decrease of threonine [100, 101], the observed deficiency of threonine proteases in T4 may have affected threonine intestinal absorption by the host. A large proportion of host dietary threonine, known as the second (or third) limiting amino acid in broilers, is predominantly used by the host to produce mucin, an important glycoprotein that preserves the integrity of intestinal mucosa and function (Qaisrani et al., 2018). With this, it is hypothesised that there could be impaired intestinal permeability in T4 broilers, which may have then contributed to overall poorer nutrient absorption, thereby affecting growth and performance. In addition, T4 in C1 has also been revealed to have differentially abundant genes for metalloproteases, a family of peptidases previously linked to overactivity in patients with irritable bowel syndrome [102, 103] Meanwhile, the elevated levels of HB metrics in T4 of C2 were unexpected given that reduction of CP in diets has commonly been associated with lower incidence of footpad lesions and better litter quality [104, 105]. However, significances in abundance among C2 groups were determined for cysteine proteases, which are proteases renowned for their involvement in virulence and their ability to induce inflammatory responses, including atopic dermatitis in humans [106, 107]. It is interesting to note that C2 groups have both higher cysteine peptidases and HB levels than C1 groups (especially C01A), also indicating a possible link between cysteine levels and HB occurrence.

The gut microorganisms participate in the metabolism and uptake of numerous nutrients and play crucial roles in preserving the integrity of the intestinal barrier, regulating the immune system, and protecting against pathogen colonisation [2]. In this study, we primarily identified the differentiation of metabolic functions involved in energy production, nucleotide metabolism, and drug transport-related pathways among treatment groups. This is in line with previous research that has shown the following: (1) Probiotic supplementation in broilers can affect vitamin biosynthesis and other energy-related metabolic activities [26]; (2) caecal microbial changes due to probiotics can affect emissions such as nitrogen or ammonia [108]; (3) antimicrobials can disrupt the nucleotide pool of bacterial cells, resulting in increased nucleotide biosynthesis and elevated central carbon metabolism [109]; and (4) by influencing the expression of microbial enzymes involved in pathways linked to nucleotide, amino acid, carbohydrate, and energy metabolism, antimicrobials could potentially steer metabolic flow, regulating bacterial proliferation or generating metabolites that affect the host [110]. Specifically, we observed a reduction of the phosphotransferase system, cofactor and vitamin metabolism, and serine and threonine modules in several GHI groups (T5 and T6) compared to T1, which are pathways involved in nutrient absorption and defence against infection in the host [111–113]. An increase in a module on methane metabolism was also detected in T3 of C1, which was also similarly reported in other studies involving probiotic use in chickens [114, 115]. This was only attributed to one KEGG module (M00345), which was detected in bin.92 UBA7160 (Lachnospiraceae). We also observed a relative decrease in drug transport-related modules in several GHI groups, namely bacitracin, lantibiotic, and PatAB transport systems. Further research is needed to confirm whether this can be explained by the similarity in pharmacological mechanisms of ionophores to bacitracin and lantibiotics, both of which are antimicrobials that also prevent cell wall synthesis and mainly act on gram-positive bacteria [116, 117].

SCFA, metabolites synthesised by caecal gut microorganisms from the breakdown of dietary fibre, play vital roles in improving metabolism facilitating nutrient digestion and absorption, thereby promoting optimal health, growth, and well-being in poultry [2]. From our analysis, we generally observed gene differentiation of SCFA modules among our treatments, wherein T5 was observed to have relatively higher gene abundance among other groups, especially in acetate production. We speculate this can be due to the effect of essential oils given in T5, which coincides with the increase of acetic and butyric acids in the caecum of broilers given dietary oregano aqueous extracts [118]. This finding, however, does not confirm if the abundance of SCFAs produced by gut microbes is optimal for gut health and performance in broilers since exorbitant amounts of SCFAs may activate the gut microbiota-brain-cell axis response, resulting in either enteritis or other metabolic syndromes [119]. Furthermore, greater amounts of propionate and butyrate acids were previously detected in birds with low feed efficiency than those with high feed efficiency [120]. Nonetheless, a higher number of EPEF + MAGs were shown to possess at least one KEGG module associated with SCFA production, compared to EPEF-MAGs, potentially indicating a contribution of SCFA production capacity of caecal gut microbiota to broiler performance.

Our study boasts several strengths, including the commercial farm set-up representing real-life poultry industrial farming, the utilisation of shotgun metagenomic sequencing data, and thorough assessments of performance characteristics. These aspects empowered us to delve into the intricate composition and functions of gut microbiota concerning GHI administration with meticulous resolution and effective control of potential confounding factors. As our sampling was limited to a single genetic line of chickens and confined to a caecal microbial study at one time point, we missed the opportunity to observe the effects of GHI on early development and its potential links to temporal and spatial shifts of the chicken gut microbiome. For instance, a previous study by Gao et al. [121] demonstrated that maturation of gut microbiota is promoted by probiotic administration while delayed by antibiotic use, highlighting the importance of broiler age in the use of supplements. Additional study into other gut compartments, timepoints, and other metagenomic features is therefore warranted. This includes a deeper investigation of other gut microorganisms, such as bacteriophages and fungi, and of other relevant microbial elements, including CRISPR-Cas systems, resistance, stress genes, and virulence genes. In addition, the future application of a multi-omics approach involving proteomics, meta-transcriptomics, and metabolomics may confirm several of our hypotheses and uncover other areas we are not able to explore. Nevertheless, we believe our research represents a novel and comprehensive comparative investigation of the metagenomic changes between the administration of ionophores and GHIs in broilers.

## Conclusion

Metagenomics has provided valuable insights into the bacterial populations in the chicken caeca, revealing differences in composition, diversity, and metabolic function influenced by various gut health strategies. This approach has also enabled us to explore the structure of the gut bacterial community in relation to key performance-related metadata. We identified several MAGs, such as *R. microfusus*, UBA 7160 species, and *Lactobacillus* species, as beneficial organisms due to their positive association with EPEF and higher capacity for metabolic functions. Such information will enhance our understanding of the highly complex relationship between gut microbes and optimal performance. It will also enable us to devise effective interventions and control strategies against enteric pathogens, which are important members of the poultry gut microbiome.

Among the gut health strategies investigated in this study, we observed that the use of probiotics B in a flock, as observed in C2, enables better bird performance. Specifically, supplementation of probiotics B in conjunction with vaccination is observed as the best GHI strategy, resulting in a similar performance to the control. However, we still observe the ionophore group to have the best performance, which is hypothesised to be due to their ability to moderate microbial diversity, resulting in more efficient capture of nutrients by the gut microbiota and, subsequently, by the host. Nonetheless, our results demonstrate supplementation of GHIs as an effective method for broiler gut modulation, with evidence of having various influences on both MAG composition and nutrient utilisation-related metabolic functions. Our data also suggests that excessive administration of GHIs may not be beneficial for performance, highlighting the importance of careful selection of GHI type and GHI combinations. These results significantly enhance our comprehension of microbiota-related metabolic pathways, offering new avenues to improve overall performance and poultry health.

## Supplementary Information

Supplementary Material 1. The summary statistics of MAGs recovered within the study.

Supplementary Material 2. Proportion-wise distribution of recovered MAGs at different taxonomic levels.

## Data Availability

Sequence data that support the findings of this study have been deposited in the European Nucleotide Archive (ENA) with the primary accession code PRJEB79352.

## Abbreviations

ADG: Average daily gain
ANOVA: Analysis of variance
BW: Bird weight
CAZymes: Carbohydrate-active enzymes
COM: Completeness (genome)
CON: Contamination (genome)
CP: Crude protein
DMRT: Duncan multiple range test
EPEF: European poultry efficiency factor
F: Finisher diet
FA: Fisher’s alpha
FCR: Feed conversion ratio
FPD: Footpad dermatitis
G: Grower diet
GC: Guanosine-cytosine
GH: Glycoside hydrolase
GHI: Gut health intervention
GLLVM: General linear latent variable model
H: Shannon’s index
HB: Hock burn
KO: KEGG orthology
LASSO: Least absolute shrinkage and selection operator
MAG: Metagenome-assembled genomes
MT: Mortality
NSP: Non-starch polysaccharides
PA: Probiotics A
PB: Probiotics B
PCA: Principal component analysis
PCoA: Principal coordinate analysis
PG: Phylogenetic gain (novelty)
PERMANOVA: Permutational multivariate ANOVA
PL: Polysaccharide lyase
R: Richness
S: Specnumber
Si: Simpson
SCFA: Short-chain fatty acids
SCG: Single-copy genes
TSS + CLR: Total sum scaling and centralised log ratio
W: Withdrawal diet
